# The Neutrophil-Lymphocyte Ratio Is Associated with Coronary Artery Calcification in Asymptomatic Korean Males: A Cross-Sectional Study

**DOI:** 10.1155/2017/1989417

**Published:** 2017-02-09

**Authors:** Su-Hyun Nam, Sung-Goo Kang, Sang-Wook Song

**Affiliations:** ^1^Department of Family Medicine, St. Vincent's Hospital, College of Medicine, The Catholic University of Korea, Suwon, Republic of Korea; ^2^Health Promotion Center, St. Vincent's Hospital, College of Medicine, The Catholic University of Korea, Suwon, Republic of Korea

## Abstract

*Introduction*. The neutrophil-lymphocyte ratio (NLR) is a significant systemic predictor of cardiovascular disease (CVD). The coronary artery calcium score (CACS) reflects coronary artery calcification and is an independent risk factor for coronary artery stenosis. In the present study, we explored the relationship between the NLR and CACS in terms of subclinical inflammation and coronary artery calcification.* Materials and Methods*. We evaluated males and females who did not have CVD, diabetes, high blood pressure, or high fasting blood sugar levels. We measured white blood cell, neutrophil, lymphocyte counts, fasting blood sugar, total cholesterol, high-density lipoprotein cholesterol, triglycerides (TG), and high-sensitivity C-reactive protein levels in blood samples. We also obtained CACSs using coronary multidetector computed tomography.* Results*. Multivariate logistic regression showed that older age was significantly associated with a higher CACS (*P* < 0.001); males had higher CACSs than females (*P* < 0.001); and the higher the TG level, the higher the CACS (*P* = 0.019). The NLR of males, but not females, was significantly associated with the CACS.* Conclusion*. An independent association between the NLR and CACS was thus evident in healthy adult males after adjusting for other CVD risk factors. Therefore, the NLR is a significant predictor of potential CVD in male subjects with subclinical atherosclerosis.

## 1. Introduction

Coronary artery disease (CAD) caused by atherosclerosis is a leading cause of adult death in developed countries [1]. In South Korea, westernization of eating habits and aging of the population are today associated with high CAD-associated morbidity and mortality. Atherosclerosis is a multifactorial disease involving several cardiovascular risk factors including dyslipidemia, high blood pressure, a high blood glucose level, and smoking. Recent studies have shown that atherosclerosis is not a passive injury caused by deposition of serum lipids and other substances on vascular walls but rather is an active inflammatory process [2]. Leukocytes are activated during inflammation, triggering the development of atherosclerosis and greatly increasing the risk of thrombus formation [3].

The neutrophil-lymphocyte ratio (NLR) (the neutrophil count divided by the lymphocyte count) is easily derived and serves as an indicator of systemic inflammation [4, 5]. The NLR is prognostic of acute coronary syndrome and is considered a much more reliable predictor than is any other predictor based on leukocyte data [6, 7]. The NLR is a marker of inflammation and is predictive of death, myocardial infarction, and coronary artery disease [6, 8, 9]. A high NLR is prognostic of atherosclerotic progression [10]. Furthermore, many epidemiological studies have shown that chronic low-grade inflammation, as indicated by the NLR, plays roles in diabetes, hypertension, metabolic syndrome, obesity, dyslipidemia, and endothelial dysfunction [11, 12].

Coronary multidetector computed tomography (MDCT) is a relatively novel method used to diagnose coronary artery disease. The coronary artery calcium score (CACS) reflects the extent of calcification of the coronary arteries. The CACS was calculated by the method of Agatston et al., yielding a total CACS that is the sum of the CACSs of each artery [13]. A higher CACS indicates a higher risk of CVD [14]. The CACS is proportional to the extent of atherosclerotic plaque and can predict coronary artery stenosis, another independent predictor of ischemic heart disease [15, 16]. Calculation of CACSs would assist in reducing the incidence of ischemic heart disease, allowing an early diagnosis of coronary artery atherosclerosis.

Today, the Framingham risk score, which considers various risk factors (age, sex, high blood pressure, the presence of diabetes, dyslipidemia, and smoking), is widely used to evaluate the risk of CVD. However, approximately half of all patients with CVD have either none or one of these risk factors [17, 18]. As inflammation is associated with atherosclerosis development and progression, we hypothesized that the NLR, considered in combination with other risk factors, aids in the prediction of CVD. Thus, we explored whether the NLR was associated with atherosclerosis measured by the CACS in healthy Korean subjects.

## 2. Materials and Methods

### 2.1. Study Population

The study subjects were 1,009 adults who underwent complete physical examinations and MDCT in the Health Promotion Center of a general hospital located in Gyeonggi-do, Korea. Of these subjects, those who had cancer, angina pectoris, acute myocardial infarction, and cerebral or peripheral vascular disease were excluded, as were those diagnosed with hypertension or diabetes or who were taking medications to treat these conditions. We performed more than two checkups on all subjects and excluded those who, on any checkup, had a systolic blood pressure >140 mmHg, a diastolic pressure >90 mmHg, and a blood fasting blood sugar level >126 mL/dL. Also, subjects with white blood cell (WBC) counts >10,000/*μ*L were excluded, because they may have had acute infectious disease. Finally, 599 subjects participated in the study.

### 2.2. Methods

#### 2.2.1. Basic Survey and Physical Examination

Before examination, we recorded any history of high blood pressure, diabetes, angina pectoris, or myocardial infarction. Additionally, we noted the medications used, smoking history, drinking history, and exercise habits. Weight and height were estimated to the nearest 100 g and 1 cm, respectively. BMI was calculated as weight (kg)/height^2^ (m^2^). Waist circumference (WC) was measured under the ribs (at the upper midpoint of the crista iliaca) to the nearest 1 cm, with each subject erect, feet 30 cm apart, and exhaling comfortably. Blood pressure was measured automatically after each subject had been seated for 20 min.

#### 2.2.2. Biochemical Tests

Blood was drawn from the brachial vein the morning after an overnight fast. We measured WBC, neutrophil, and lymphocyte counts and the blood levels of fasting glucose (FBG), total cholesterol, high-density lipoprotein cholesterol, triglycerides (TG), aspartate transaminase, alanine transaminase (ALT), gamma-glutamyl transpeptidase (a-GTP), creatinine, high-sensitivity C-reactive protein, uric acid, calcium, phosphate, and albumin.

#### 2.2.3. CACS

CACSs were calculated from the heart CT scans obtained using the aid of a 64-slice MDCT scanner (Sensation 64; Siemens, Erlangen, Germany). Scans were obtained after at least 6 h of fasting, with caffeine intake prohibited. If the heart rate of a subject who was not on a beta-blocker was >65 beats per min, we sought to lower the heart rate by giving atenolol (25–75 mg) 1 h before scanning. Using an autoimpregnator, we injected 60–70 mL nonionic contrast medium (Ultravist 370, Schering, Germany) followed by saline (40 mL) into the ulnar artery. We electrocardiographically synchronized the heart CT and calculated each CACS via reconstruction (Wizard; Siemens, Erlangen, Germany). CACS was calculated using the method of Agatston. The coronary artery was divided into four segments, and the scores of each segment were calculated and summed. We used the extent of pigmentation to calculate each CACS (0, >0).

#### 2.2.4. Statistics

Data were compared using Statistical Package for the Social Sciences software, version 18.0 (SPSS Inc., Chicago, IL, USA). We compared between-group parameters using the *t*-test. To afford a 95% predictive power of an odds ratio (OR) of 2 at the 5% significance level, the two-tailed *t*-test indicated that 502 subjects were required [19]. ORs and 95% confidence intervals (CIs) were calculated by multivariate logistic regression. A *P* value <0.05 was regarded as statistically significant.

#### 2.2.5. Ethics Statement

This study was implemented in accordance with ethical and safety guidelines upon the approval of the Institutional Review Board in The Catholic University of Korea, St. Vincent's Hospital (IRB approval number: VC15RISI0068). The study was exempted from the written informed consent to participants because we reviewed the health screening data and medical record retrogradely. The IRBs approved this consent procedure.

## 3. Results

### 3.1. General Subject Characteristics

Of the 599 subjects, 473 (79%) had a CACS of 0 and 126 (21%) a CACS > 0. The average age of the latter group was 56.06 ± 8.99 years, significantly higher than that of the CACS = 0 group (49.09 ± 56.06 years; *P* < 0.001). In the CACS > 0 group, WC, systolic blood pressure, and levels of ALT, GTP, TC, and TG were significantly higher than those of the CACS = 0 group. The WBC count and NLR did not differ significantly between the groups (Table 1).

### 3.2. The Relationship between the CACS and CVD Risk Factors in All Subjects

Multivariate logistic regression was used to explore the relationships between the CACS and factors that might affect the CACS (Table 2). Aging was an independent risk factor for an elevated CACS; as subject age increased, the CACS rose significantly (*P* < 0.001). Males had higher CACSs than those of females, and, as the TG level increased, the CACS increased significantly (*P* = 0.019) (Table 2)

### 3.3. The Relationship between the CACS and CVD Risk Factors in Males

**Figure 1:**
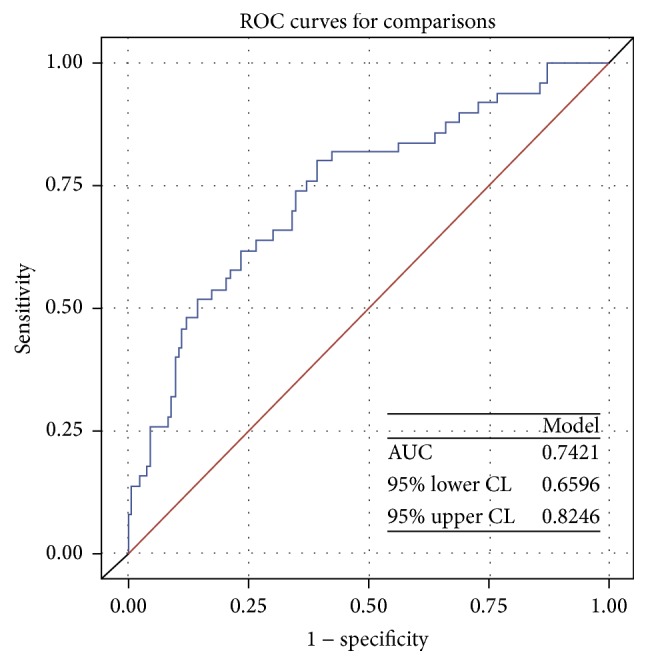
The graphs represent area under the ROC curve (AUC) of NLR in predicting CAC in men. Model was adjusted with age, smoking, BMI, WC, SBP, DBP, WBC, Hb, albumin, FBS, TC, HDL-C, TG, AST, ALT, rGTP, Cr, Uric acid, Ca, P, Ca/P ratio, and hs-CRP.

We used multivariate logistic regression to evaluate 397 males in terms of the relationships between the CACS and factors that might affect the CACS. The CACS increased significantly (*P* < 0.001) with the age of healthy asymptomatic males and was also significantly associated with elevated systolic blood pressure (*P* = 0.039). Of the hematological factors evaluated, the NLR was found to be an independent risk factor for an elevated CACS; the CACS increased significantly with the NLR (*P* = 0.045). Of the lipid-related factors, the higher the TG level, the higher the CACS (*P* = 0.022) (Table 3). After adjusting other factors, area under the ROC curve of NLR in predicting CAC was 0.74 (95% CI = 0.66–0.82) (Figure 1).

### 3.4. The Relationship between the CACS and CVD Factors in Females

Of all 136 women, age (*P* < 0.001), body mass index (*P* = 0.011), and current smoking status (*P* = 0.002) independently and significantly affected the CACS. No significant relationship was evident between the NLR and CACS in females (Table 4).

## 4. Discussion

We explored the relationship between the NLR and the CACS to determine if the NLR (a measure of subclinical inflammation) predicted the extent of coronary artery calcification in asymptomatic subjects. For healthy males (but not females), an increase in the NLR was correlated independently with an increase in the CACS.

Inflammation plays critical roles in many coronary artery diseases [20]. Atherosclerosis is a complex of inflammatory diseases [21]. Leukocytes contribute substantially to progression of inflammation [22], and several prospective studies have found positive correlations between leukocyte (total and differential) counts and the development of CVD in both adult males and females [23, 24]. The NLR is a well-recognized biomarker of inflammation [25]. When the WBC count is within the normal range, a higher NLR is indicative of a greater risk of atherosclerotic disease [6]. The NLR is associated with other clinical inflammatory markers. Balta et al. reported that there were moderate positive correlations between carotid intima media thickness value, C-reactive protein, and NLR in patients with Behçet disease [26]. Unlike many other inflammatory markers, the NLR is inexpensive and readily available and it provides additional risk stratification beyond conventional risk scores [27]. However, NLR values may be changed by medications. Fici et al. reported that nebivolol improved NLR to a greater extent than metoprolol in patients with hypertension [28].

Balta et al. found that increased NLR may play a role not only in the pathogenesis of coronary artery disease but also in the pathophysiology of coronary artery ectasia [29]. Additionally, the NLR is associated with both the risk and severity of CVD [30]. Shen et al. found that the NLR significantly predicted long-term mortality in 551 STEMI patients who underwent early cardiac revascularization (hazard ratio 2.27, 95% CI 1.32–4.29; *P* = 0.002) [31]. Wang et al. performed a meta-analysis of 10 cohort studies on patients who underwent angiography or cardiac revascularization; the relative risk (RR) of all-cause mortality in the high-NLR group was 2.33 (95% CI 1.88–2.88), and the RR of a cardiovascular event was 1.89 (95% CI 1.88–2.88). Thus the NLR significantly predicted both all-cause mortality and cardiovascular events in patients with CVD [32].

Coronary artery calcification is closely associated with coronary artery disease [33]. The CACS reflects both the extent and distribution of coronary artery calcification, which reliably predicts the CVD risk [34–36]. As calcium deposits increase, so does the CVD risk [37]. One systematic study found that when the CACS was 0, the prevalence of CVD was 0.56%, and the negative predictive value 99% (thus very high) [38]. When the CACS was <100, the risk of an angiographic diagnosis of significant coronary artery stenosis (>50%) was <3% [39]. In a 3-year study on 2,000 asymptomatic adults, a CAC > 0 increased the risk of CVD 10.5-fold in males and 2.6-fold in females [40].

Turkmen et al. evaluated 56 patients (34 males, 22 females) with end-stage renal disease who were on dialysis for >6 months. Except for in those with acute infections, autoimmune diseases, acute heart failure, or CVD, the NLR and CACS were significantly correlated (*r* = 0.3, *P* = 0.02) [41]. Also, in a study of 290 patients with type 2 diabetes mellitus, those with NLRs ≤2.05 had lower CACSs and a lower incidence of obstructive coronary artery disease than did those with NLRs >2.05 [42]. Park et al. used multivariate logistic regression to show that a high NLR was independently associated with an elevated CACS in 849 Koreans [43]. The cited work included patients with hypertension and diabetes, and the data were not analyzed by sex. We excluded patients with diabetes or hypertension and showed that the NLR was correlated independently with the CACS (after adjustment for other CVD risk factors) of healthy adult males with FBG levels <126 mg/dL, systolic blood pressure <140 mmHg, and diastolic blood pressure <90 mm Hg. Therefore, the NLR can be used to predict CVD not only in patients at high risk of CVD (such as those with diabetes, hypertension, and chronic renal failure) but also in asymptomatic healthy adult males.

We found that the TG level was independently associated with the CACS, in agreement with the data of Cao et al. [44]. Tirosh et al. evaluated 13,953 healthy adult males aged 26–45 years for 5 years and found a strong correlation between the TG level and the development of CVD [45]. A meta-analysis of studies on adults of mean age of 56.6 years, over a 12-year follow-up, found that the CVD risk was 1.8-fold greater in those with the highest 20% of TG levels, compared with those with the lowest 20% [46]. Also, in the Framingham study, the risk of CVD was 2-fold higher in those with TG levels of 250–400 mg/dL than in those with TG levels of 50–100 mg/dL [47]. The Copenhagen study recruited middle-aged males (>54 years of age). In those with TG levels >142 mg/dL, the adjusted RR of CVD was 2.2, significantly higher than the RRs of those with lower TG levels [48]. Tirosh et al. showed that elevated TG levels correlated significantly with the body mass index, the extent of physical activity, and breakfast habits [45]. Therefore, lifestyle-mediated management of TG levels will greatly lower the CVD risk.

We found a close association between female smoking and the CACS. Lehmann et al. reported that current smoking was an independent risk factor for a CACS > 0, and that the time until reaching a CACS > 0 was approximately 10 years less in current versus former smokers [49]. Smoking lowers the availability of nitrogen oxide, triggering endothelial and vasomotor dysfunction, activating leukocyte-endothelial cell interactions, triggering leucocyte recruitment, increasing the levels of proatherogenic substances by elevating proinflammatory cytokine levels, and triggering oxidative lipid modifications, culminating in atherosclerosis [50].

Our study had a few limitations. First, we considered only present smoking status; we did not record pack-years, and we did not consider alcohol ingestion, self-reporting bias, or stress associated with smoking. Thus, we found no relationship between smoking and the CACS in males. Had we considered the above factors, such an association might have become apparent. Second, our work was cross-sectional in nature; caution is thus appropriate when seeking to identify causative relationships.

In conclusion, we found an independent correlation between the NLR and the CACS in asymptomatic Korean adult males. The NLR can be used to predict CVD in subjects with subclinical atherosclerosis.

## Figures and Tables

**Table 1:** Baseline characteristics of study populations.

	CACS = 0	CACS > 0	*P* value
Age (year)	49.04 ± 8.46	56.06 ± 9.00	<0.001
Sex			<0.001
Male	295 (74.3%)	102 (25.8%)	
Female	178 (88.1%)	24 (11.9%)	
Smoking			0.912
Nonsmoker	337 (78.7%)	91 (21.3%)	
Smoker	136 (79.5%)	35 (20.5%)	
BMI (kg/m^2^)	23.91 ± 2.70	24.62 ± 3.20	0.011
WC (cm)	84.31 ± 8.41	86.88 ± 8.42	0.003
SBP (mmHg)	119.43 ± 11.37	122.27 ± 10.10	0.007
DBP (mmHg)	72.68 ± 8.32	72.92 ± 7.54	0.761
WBC (10^3^/*μ*L)	5.95 ± 1.58	6.24 ± 1.73	0.086
Neutrophils (10^3^/*μ*L)	53.07 ± 8.78	52.19 ± 8.16	0.315
Lymphocytes (10^3^/*μ*L)	36.28 ± 7.70	36.90 ± 7.88	0.435
Neutrophil-lymphocyte ratio	1.59 ± 0.63	1.53 ± 0.60	0.416
Hb	14.78 ± 1.55	15.05 ± 1.37	0.059
Albumin	4.54 ± 0.29	4.47 ± 0.39	0.084
FBS (mg/dL)	92.03 ± 9.19	95.83 ± 10.77	<0.001
TG (mg/dL)	124.83 ± 72.68	141.77 ± 72.70	0.020
TC (mg/dL)	201.98 ± 35.47	213.47 ± 64.24	0.008
HDL-C (mg/dL)	47.24 ± 11.00	45.40 ± 9.85	0.090
AST (IU/L)	22.06 ± 12.32	24.29 ± 11.54	0.068
ALT (IU/L)	25.49 ± 20.48	30.09 ± 24.59	0.032
rGTP (IU/L)	32.78 ± 33.16	44.85 ± 52.53	0.015
Cr (mg/dL)	0.84 ± 0.16	0.97 ± 0.74	0.061
UA (mg/dL)	5.58 ± 1.45	5.78 ± 1.37	0.182
Ca	9.09 ± 0.35	9.96 ± 8.99	0.317
P	3.27 ± 0.47	3.28 ± 0.46	0.735
Ca/P Ratio	2.84 ± 0.42	3.13 ± 3.27	0.360
hsCRP (mg/dL)	0.15 ± 0.35	0.20 ± 0.29	0.195

**Table 2:** Odds ratios and 95% intervals for CACS in men and women in multivariate analysis.

Variables	OR	95% CI		*P* value
		Lower	Upper	
Age (year)	1.166	1.101	1.234	<0.001
Female	0.048	0.010	0.226	<0.001
Current smoker	0.601	0.221	1.633	0.318
BMI (kg/m^2^)	1.207	0.946	1.539	0.130
WC (cm)	0.934	0.854	1.022	0.136
SBP (mmHg)	1.041	0.987	1.098	0.135
DBP (mmHg)	0.961	0.895	1.032	0.271
WBC (10^3^/*μ*L)	0.960	0.710	1.299	0.793
Neutrophils (10^3^/*μ*L)	1.062	0.936	1.205	0.353
Lymphocytes (10^3^/*μ*L)	1.101	0.938	1.279	0.208
Neutrophil-lymphocyte ratio	2.209	0.222	21.938	0.499
Hemoglobin	0.652	0.439	0.967	0.034
Albumin	0.176	0.031	0.995	0.049
FBS (mg/dL)	0.993	0.951	1.037	0.760
TC (mg/dL)	0.999	0.990	1.009	0.854
HDL-C (mg/dL)	1.001	0.959	1.044	0.975
TG (mg/dL)	1.008	1.001	1.014	0.019
AST (IU/L)	1.076	1.008	1.147	0.027
ALT (IU/L)	0.997	0.960	1.035	0.870
rGTP (IU/L)	0.994	0.981	1.008	0.415
Cr (mg/dL)	2.261	0.422	12.119	0.341
UA (mg/dL)	1.077	0.794	1.461	0.635
Ca	1.912	0.362	3.388	0.315
P	0.642	0.008	5.640	0.843
Ca/P ratio	0.759	0.006	9.762	0.910
hsCRP (mg/dL)	1.078	0.340	3.411	0.899

**Table 3:** Odds ratios and 95% intervals for CACS in men in multivariate analysis.

Variables	OR	95% CI		*P* value
		Lower	Upper	
Age (year)	1.154	1.077	1.237	<0.001
Current smoker	0.499	0.159	1.565	0.233
BMI (kg/m^2^)	1.024	0.735	1.428	0.887
WC (cm)	0.946	0.839	1.066	0.362
SBP (mmHg)	1.076	1.004	1.153	0.039
DBP (mmHg)	0.924	0.846	1.010	0.083
WBC (10^3^/*μ*L)	0.874	0.600	1.275	0.485
Neutrophils (10^3^/*μ*L)	0.956	0.845	1.083	0.479
Lymphocytes (10^3^/*μ*L)	1.148	0.966	1.365	0.118
Neutrophil-lymphocyte ratio	7.464	1.047	53.212	0.045
Hemoglobin	0.584	0.343	0.993	0.047
Albumin	0.368	0.052	2.602	0.317
FBS (mg/dL)	0.970	0.921	1.022	0.257
TC (mg/dL)	1.004	0.994	1.014	0.445
HDL-C (mg/dL)	0.976	0.922	1.020	0.416
TG (mg/dL)	1.011	1.001	1.020	0.022
AST (IU/L)	1.095	1.007	1.189	0.033
ALT (IU/L)	0.984	0.940	1.030	0.487
rGTP (IU/L)	0.991	0.974	1.007	0.256
Cr (mg/dL)	2.240	0.506	9.918	0.288
UA (mg/dL)	0.988	0.693	1.409	0.948
Ca	3.376	0.241	47.210	0.366
P	10.477	0.028	39.128	0.437
Ca/P ratio	8.464	0.241	47.210	0.507
hsCRP (mg/dL)	3.251	0.385	27.422	0.279

**Table 4:** Odds ratios and 95% intervals for CACS in women in multivariate analysis.

Variables	OR	95% CI		*P* value
		Lower	Upper	
Age (year)	1.303	1.138	1.492	<0.001
Current smoker	99.872	5.450	1830.127	0.002
BMI (kg/m^2^)	1.735	1.135	2.654	0.011
WC (cm)	0.907	0.822	1.001	0.052
SBP (mmHg)	1.008	0.922	1.101	0.867
DBP (mmHg)	0.943	0.823	1.080	0.394
Neutrophils (10^3^/*μ*L)	1.204	0.954	1.519	0.117
Lymphocytes (10^3^/*μ*L)	0.798	0.596	1.067	0.128
Neutrophil-lymphocyte ratio	0.010	0.000	2.585	0.104
Hemoglobin	0.748	0.391	1.432	0.381
FBS (mg/dL)	1.052	0.972	1.139	0.211
TC (mg/dL)	1.012	0.989	1.036	0.314
HDL-C (mg/dL)	0.977	0.905	1.055	0.553
TG (mg/dL)	0.989	0.977	1.002	0.095
AST (IU/L)	0.253	0.728	1.087	0.253
ALT (IU/L)	1.155	0.988	1.350	0.071
rGTP (IU/L)	0.975	0.926	1.027	0.348
UA (mg/dL)	0.567	0.239	1.344	0.198
Ca	6.326	0.291	12.826	0.167
P	0.163	0.000	13.897	0.694
Ca/P ratio	0.216	0.000	17.147	0.791
